# “*It is not possible for me to have diabetes*”–Community Perceptions on Diabetes and Its Risk Factors in Rural Purworejo District, Central Java, Indonesia

**DOI:** 10.5539/gjhs.v6n5p204

**Published:** 2014-06-11

**Authors:** Cahya Utamie Pujilestari, Nawi Ng, Mohammad Hakimi, Malin Eriksson

**Affiliations:** 1 Department of Public Health and Clinical Medicine, Epidemiology and Global Health, Umea University, Sweden

**Keywords:** content analysis, diabetes, focus group, health behaviour, Indonesia, perception, qualitative methods, unrealistic optimism

## Abstract

Accumulating evidence suggests that negative perceptions towards diabetes can limit the management and prevention of the disease. The negative perceptions towards diabetes are prevalent in many different settings, especially among rural communities. Few qualitative studies have been performed to understand how the community views diabetes and its associated risk factors. This study aimed to explore general community perceptions of diabetes and its risk factors in rural Indonesia. A total of 68 participants were recruited to 12 focus group discussions (FGDs) comprised of different age groups and sexes. The FGDs were conducted in six villages in rural Purworejo District, Central Java, Indonesia, from 2011 to 2012. All FGDs were recorded and transcribed. Qualitative content analysis was performed to describe and analyse how the rural community perceived diabetes and its risk factors. Diabetes was perceived as *a visible and scary sugar disease*, and the affected individuals themselves were blamed for getting the disease. Recognised as ‘sugar’ or ‘sweet-pee’ disease with terrifying effects, diabetes was believed to be a disease with no cure. The participants seemed to have an *unrealistic optimism* with regards to the diabetes risk factors. They believed that diabetes would not affect them, only others, and that having family members with diabetes was necessary for one to develop diabetes. Our findings demonstrate that rural communities have negative perceptions about diabetes and at the same time individuals have unrealistic optimism about their own risk factors. Understanding how such communities perceive diabetes and its risk factors is important for planning prevention strategies. Health messages need to be tailored to health-related behaviours and the local culture’s concepts of diseases and risk factors.

## 1. Introduction

Diabetes mellitus is one of the fastest growing chronic non-communicable diseases (NCDs) and has become a global epidemic. The World Health Organization (WHO) estimates that a total of 171 million people were diagnosed with diabetes worldwide in 2000 and that this number will increase to 366 million in 2030. In Indonesia, the number of diabetes cases doubled from 4.5 million in 1995 to 8.4 million in 2000. In the absence of primary prevention strategies in the country, this number is projected to increase up to 21 million in 2030, with the majority developing type 2 diabetes mellitus (T2DM) (Wild et al., 2004).

Evidence shows that T2DM can be effectively prevented through lifestyle (behavioural) risk factor interventions (Li et al., 2008; Lindström et al., 2006; Wing, 2010). The major lifestyle risk factors related to T2DM include overweight and obesity, tobacco use, physical inactivity, low fruit and vegetable intake, and a diet high in salt and fat (Danaei et al., 2009; Lyssenko et al., 2008). Indonesia faces increasing rates of many of the risk factors involved in NCDs and has seen increasing trends for overweight and obesity, high blood pressure, and high cholesterol from 1980 to 2008 (WHO, 2011). There are a number of studies on the prevalence of NCDs and associated risk factors in Indonesia (Ashraf et al., 2009; Bich et al., 2009; Ramachandran et al., 2012; Shaw et al., 2010; Whiting et al., 2011), but qualitative studies exploring community views on NCD-related illnesses and health effects are almost non-existent.

There are numerous qualitative studies describing how diabetic patients view their disease and the barriers facing diabetes management (Al-Qazaz et al., 2010; Greenfield et al., 2011; Macaden & Clarke, 2006; Naithani et al., 2006; Pera, 2011; Yamakawa & Makimoto, 2008). However, only a few qualitative studies have been conducted to understand how the communities in general view diabetes and its associated risk factors. A study focussing on two Iranian cities (Isafahan and Tehran) found that the participants viewed diabetes as ‘blackness’, ‘end of romances’ and a ‘gradual death’ (Abdoli et al., 2012). In a study from Kenya, lack of accurate knowledge regarding the causes and effects of diabetes were found in more than 70% of the respondents (Maina et al., 2010).

Another study on diabetes knowledge and perceptions was conducted in the Appalachian population in West Virginia in the US (Tessaro et al., 2005). The Appalachian region was described as a lower socio-economic area with many social and health disparities contributing to a high rate of diabetes, and the study showed that participants had poor perceptions of diabetes risks beyond those of ‘heredity’ (a family history of diabetes), ‘inactivity (laziness)’ and ‘lack of self-control (eating too much sugar) (Tessaro et al., 2005). The authors highlight the need for tailoring specific educational interventions to low socioeconomic communities as those common beliefs often create ‘blame’ and ‘guilt’ along with the diagnosis (Tessaro et al., 2005). A lack of accurate knowledge about T2DM might be even more prevalent among people in low- and middle-income countries where T2DM is not yet viewed as an important health issue. No qualitative studies about the perceptions of T2DM and its associated risk factors among the general population have been conducted in Indonesia.

Understanding community views and perceptions in regards to disease and risk factors is essential to design and achieve successful health promotion strategies. The *Health Belief Model* states that the actions people take to maintain their health depend on how they perceive the threat of the disease (Glanz et al., 2002). Perception is defined as “our capacity to perceive the world by means of our sense organs” (Fish, 2010). If people perceive that they are susceptible to a disease and are likely to suffer serious consequences from it, then people tend to take action to prevent the disease. This study aims to explore community perception of diabetes and its risk factors in rural areas in Purworejo District, Central Java, Indonesia, and these perceptions are discussed in terms of the Health Belief Model.

## 2. Methods

### 2.1 Study Design & Sample

This study used a qualitative design and was conducted in Purworejo District, Central Java, Indonesia. Purworejo is located in the southern part of Java Island and had a population of 898,631 (Purworejo, 2013). Purworejo has 16 sub-districts (494 villages) and about 2,000 to 3,000 individuals live in each village. The majority of the areas in Purworejo are rural settlements with low population density. Purworejo’s economy is heavily dependent on agricultural and livestock trading activities.

This study utilized focus group discussions (FGDs) to their suitability for exploring community perceptions (Dahlgren et al., 2007). A total of 12 FGDs were conducted from July 2011 to February 2012. The participants were purposefully selected from a total of six rural villages that represented different geographical areas, including highland (mountainous), lowland (coastal), and suburban areas (close to the district centre). We conducted two FGDs in each village and purposefully decided on the compositions and inclusion criteria of each group.

The selected groups represented men and women in different age groups (Table 1), and this stratification aimed to create free and comfortable environments for the discussion groups. The idea was to encourage younger people and women to discuss their ideas freely and actively without worrying about what older people or those of the opposite sex would think.

**Table 1:** Number of participants and villages in the FGDs by sex and age

Village	No of group	Participants						Total

		20-34 y		35-54 y		55+ y		

		Men	Women	Men	Women	Men	Women	
**Pakem (Highland)**	2	5					6	11
**Sokowaten (Lowland)**	2			6		6		12
**Watukuro (Satellite)**	2		6		7			13
**Keduren (Highland)**	2	6	4					10
**Mlaran (Lowland)**	2			5	6			11
**Candisari (Satellite)**	2					6	5	11

**Total**	**12**	11	10	11	13	12	11	**68**

### 2.2 Procedure and Process

The main researcher (CP) conducted all of the FGDs with the help of a local research assistant who was a native Javanese speaker. The research assistant worked together with the village leader to recruit and invite five or six participants to each FGD. The village leader suggested the study participants based on his or her knowledge of the participant’s willingness to discuss and participate in the FGD. Participants were invited to the village hall or a house of one of the volunteering participants for the discussions. The discussions lasted for approximately 60 to 90 minutes and were recorded using a digital voice recorder. Snacks and a small monetary incentive were given to the participants as reimbursement for the time they spent in the FGD.

The FGDs were divided in two sections. Section one started with the open-ended question, “What have you heard about diabetes?” Participants were encouraged to share and discuss their opinions, perceptions, knowledge, and experiences of diabetes. In section two, we distributed a set of picture cards to each participant showing 12 risk factors for diabetes. The risk factors included age, fast food, family history with diabetes, overweight and obesity, smoking, low physical activity, low fruit and vegetable consumption, stress, race/ethnicity, anti-hypertensive medication, x-rays, and pets. The last two cards were included as false examples of risk factors. The participants were asked to divide the cards into those that they believed were or were not risk factors for diabetes and were then asked to give arguments for their choices. However, after having conducted two FGDs this method was found to be too time consuming and the participants had difficulties in deciding how to divide and formulate arguments for the cards. For the subsequent FGDs, we decided to distribute the 12 cards (including the false examples) to be shared among the participants. Each participant received two or three cards and was asked to give arguments based on the cards they had. Other participants were encouraged to argue and join the discussion.

The FGD guide was developed in English and was translated into Indonesian language (Table 2). At the end of the discussion, the participants were asked to fill in a form consisting of questions on demographic characteristics, family history of diabetes, self-rated health, and their perception of their personal risk of developing diabetes.

**Table 2:** Focus group discussion guide in English

Section one (diabetes in general)	Section two (diabetes risk factors)
1. What have you heard about diabetes? What kind of disease it is?	6. What do you think about the picture in the cards?
2. Is it dangerous to have diabetes?	7. Is that picture somehow related with diabetes?
3. Is diabetes common in your community? (Optional)	8. What in your opinion causes diabetes?
4. Is there anyone in your family that has diabetes? Would you like to share about it? (Optional)
5. How in your opinion would life be affected if you had diabetes? (Optional)	

### 2.3 Data Analysis

All of the FGDs were transcribed word for word in their original language (Bahasa Indonesia and/or Javanese). The native Javanese assistant later translated all sections in Javanese into Bahasa Indonesia to ensure the correct meaning before the transcripts were analysed.

The transcripts were analysed using qualitative content analysis as described by Graneheim and Lundman (2004). The analysis began by finding meaning units in the transcribed text that were relevant to the research questions, i.e., perceptions about diabetes and perceptions about diabetes risk factors. The meaning units were then developed into condensed meaning units, which were then coded. In the next step, the codes were clustered according to the two content areas of perception of diabetes and perception of risk factors. These two content areas were analysed separately.

Within each content area, specific patterns arose during the coding process. We determined sub-categories according to issues that were repeatedly emphasized by the groups and by sorting codes due to their commonality. The sub-categories were then sorted into categories. In the last step, themes were created to link the underlying meanings in the categories. All of the analyses were conducted in English and shared with the other co-authors for joint analysis.

We analysed the data separately for men and women in different age groups to look for possible differences in perception across the groups. The results are presented according to content area. Table 3 illustrates the process of moving from meaning unit to codes, and Table 4 illustrates the process of generating categories from the codes.

**Table 3:** Audit trail example from text to code clusters in young men

FGD content analysis	Men – young FGD
**Meaning unit**	I think smoking is not related to sugar disease but there must be small part that can affect sugar disease; most smokers, including me, we usually deny
**Condensed meaning unit**	Smoking is related to diabetes ‘but small part’; smokers deny bad effect of smoking;
**Codes**	Smoking only partly related to diabetes
	Smokers deny bad effect of smoking
	Aware of healthy lifestyle but ignoring
**Codes cluster**	**Diabetes content area:**
	-
	**Risk factors content area:**
	Smoking only partly related to diabetes
	Smokers deny bad effect of smoking
	Aware of healthy lifestyle but ignoring

**Table 4:** Audit trail example from codes to themes in the content area ‘diabetes perception’ in women

FGD analysis	Women			
**Codes**	Diabetes is a disease among wealthy family,	‘Dry sugar’ patients often hospitalized	Unhealed wound caused amputation	Diabetes and heart disease is related,
	Diabetes is diseases of prosperity	Money is needed to survive with diabetes	It is scary to have unhealed wound	Diabetes and heart disease cause death
	Poor people rarely have sugar disease	Diabetes care is expensive	Many people has diabetes ended up with death	Bad diet cause complications
	Wealthy people tend to have sugar disease	Diabetes treatment cause family economy burden	We do not know about diabetes until we get sick	Sugar disease invite other diseases
	Low economy people tend to be careless about their body (disease)	Wealthy people can directly find health care	Diabetes recognized when having wound,	Uncertainty: diabetes developed to heart disease (?)
**Sub-categories**	**Disease of prosperity**	**Diabetes care requires money**	**Diabetes has unrecognized symptom that may end up with amputation and death**	**Diabetes cause complications**
**Categories**	A disease for ‘the wealthy’		A silent disease with ‘terrifying’ effects	
**Theme**	**A visible and scary sugar disease that can be blamed by modern lifestyle**			

### 2.4 Trustworthiness

The trustworthiness of the study was ensured by several criteria such as truth-value, applicability, consistency, and neutrality (Lincoln & Guba, 1985). To enhance the truth-value or credibility, we used investigator triangulation in terms of expertise and cultural understanding. This allowed us to evaluate the issues from different perspectives (Dahlgren et al., 2007). All four of the researchers work within the field of public health and were continuously involved in all stages in this research. The first author (CP) is a researcher with a nursing background and an interest in chronic NCDs; the second and third authors (NN and MH) are public health scientists with medical doctor backgrounds and experience in NCD research; and the last author (ME) is a social worker with a research interest in social determinants of health. CP, NN, and MH are Indonesian, and both CP and MH have a good understanding of the Javanese culture. ME is a Swede with an outsider’s perspective on the research topic and setting. During data collection, ME attended two FGDs (one with men and one with women) and the participation enriched ME’s perspectives on the study setting, the participants, and the discussions flow.

The selected villages, which represented different areas in rural Purworejo, enhanced the applicability or generalizability. Having the first author conduct all of the FGDs ensured the consistency or reliability of the study. The first author and the assistant have been trained in qualitative data collection. Additionally, clear researchers notes and a detailed audit trail allowed us to enhance the overall trustworthiness of this study (Dahlgren et al., 2007).

### 2.5 Ethical Considerations

The study received ethical approval from the ethical boards of the Faculty of Medicine, Gadjah Mada University, Indonesia. We provided detailed information about the study to all participants before they were asked to sign informed consent provided prior to the FGDs.

## 3. Results

Sixty-eight participants between the ages of 20 and 82 years, with an equal number of men and women, participated in the FGDs. More than half of the participants had finished junior high school. Most of the female participants were housewives and most of the men were farmers or retirees. Thirteen out of the 68 participants reported that they had at least one family member with diabetes. When asked to rate their own health, more than 50% of the participants answered that they were in good health. About 44% of the men reported poor health, but only 26% of the women reported poor health. Most participants believed that they were not at risk of having diabetes, only 21% of the men and women thought that they were at risk. The detailed demographic and health characteristics of the participants are presented in Table 5.

**Table 5:** FGD participants’ demographic and health characteristics

Variable	Men (N=34) n (%)	Women (N=34) n (%)
**Education**		
No formal education	1 (3)	3 (9)
Finished elementary school	4 (12)	2 (6)
Finished junior high school	11 (32)	11 (32)
Finished senior high school	16 (47)	10 (29)
Finished academy or university	2 (6)	4 (12)
**Occupation**		
Farmers	12 (36)	2 (6)
Non-farmers	21 (64)	32 (94)
**Having family with diabetes**		
Yes	4 (12)	9 (26)
No	28 (82)	17 (50)
Don’t know	2 (6)	8 (24)
**Self rated health**		
Very good	2 (6)	5 (15)
Good	17 (50)	20 (59)
Poor	15 (44)	9 (26)
**Are you at risk of diabetes?**		
Yes	7 (21)	7 (21)
No	21 (62)	26 (76)
Don’t know	6 (18)	1 (3)

Our findings regarding perception about diabetes (content area 1) and perception about diabetes risk factors (content area 2) are summarized into themes. We found slight differences between age groups and gender groups in some of the categories. The themes and categories for each content area are summarized in Table 6.

**Table 6:** Theme and categories in each content area

Content areas	Themes	Categories
**1. Diabetes perception**	A visible and scary sugar disease that can be blamed by modern lifestyle	A ‘dry’ and ‘wet’ ‘sugar’ disease
		A visible disease
		A silent disease with ‘terrifying’ effects
		A disease with ‘no’ cure
		A disease for ‘the wealthy’
**2. Diabetes risk factors perception**	Unrealistic optimism in diabetes risk factors perception	Heredity and sugar is the main risk
		‘Modern’ lifestyle is a risk
		Older age increases risk
		Ambiguity in smoking risk

### 3.1 Content Area 1, Perception about Diabetes: “A Visible and Scary Sugar Disease that can be Blamed by Modern Lifestyle”

The overall theme identified within this content area was that diabetes is viewed as “a visible and scary sugar disease that can be blamed by modern lifestyle”. This theme was developed based on five identified categories as described below.

#### 3.1.1 A ‘Dry’ and ‘Wet’ Sugar Disease

The participants referred to diabetes as ‘sugar disease’ or ‘sweet-pee disease’. These terms were more familiar to the community, and were used to refer to the *cause* (‘sugar disease’ caused by eating sugar) and the *symptom* (‘sweet-pee disease’ when ants are attracted to one’s urine) of the disease. Unless there was a family member or close neighbour with diabetes, most participants were not familiar with the term ‘diabetes’. Some groups mentioned that they believed *sweet-pee disease* to be the early stage of the disease. Once the disease manifested with wounds that are considered a more severe stage of the disease, it was called *sugar disease*.

“I think that diabetes and sugar disease are different…. What I know is that sweet-pee disease is not as dangerous as sugar disease” (Men – young group)

“Diabetes is a fancy (lavish) name for me. All I know is just sugar disease…” (Men – adult group)

The participants classified diabetes according to the wound characteristics and the appearance of the patients. Two types of sugar disease mentioned were *wet-sugar* and *dry-sugar* disease. The participants characterised wet-sugar disease patients as “having watery wounds that are difficult to heal”, and dry-sugar disease patients were described as “those who lose weight and become skinny”.

“Sugar disease has two types…dry-sugar and wet-sugar…. Wet-sugar is when wounds have a difficult time healing, but the patients usually continue to ‘look healthy’. Dry-sugar is when the wound dries out… but leaves a mark…and usually they become skinny…they lose their weight” (Women – young group)

#### 3.1.2 A Visible Disease

Sugar disease and sweet-pee disease were described as being commonly observed in the participants’ communities. Some of the FGD participants were themselves diabetics or had a family member with diabetes. Even though most participants were not familiar with the term ‘diabetes’, they were aware of the existence of the disease, and most participants were able to describe the ‘symptoms’ that a diabetic usually has. Interestingly, the participants seemed to have become accustomed to the disease, and this in a way might have decreased the awareness of the seriousness of having diabetes.

“Nowadays diabetes is like a…common disease, many people have it, hehehe…(smiling) we used to be worried before…but now…diabetes is…common…” (Women - old group)

#### 3.1.3 A Silent Disease with ‘Terrifying’ Effects

Most of the participants stated that diabetes is a *silent disease* because it is difficult to distinguish its symptoms. Frequent urination at night and sweet-pee (recognized by ants being attracted to the urine) was described as commonly perceived diabetes ‘symptoms’ in the community. Participants stated that even a medical doctor could not easily recognize diabetes symptoms or complications.

“My sister once had blurred vision, she checked with the eye doctor (ophthalmologist) but he had no answer… suddenly her leg became wounded and it has been difficult to heal …” (Men – adult group)

Participants consistently mentioned unhealed wounds and amputations as terrifying effects that can lead to death among diabetes patients. Several groups mentioned that once diabetes “attacked” the patient should be ready for death.

“Sugar disease is a deadly disease because when wounds occur they difficult to heal” (Women – adult group)

“If someone has sugar disease, they must be ready for death…” (Men - young group)

The participants thought that sugar disease was closely related to heart diseases (i.e. cardiovascular disease) because both diseases were seen to appear concurrently. The participants also believed diabetes preceded other diseases.

“It is usually hypertension, heart attack, and stroke…(diabetes complications)… but I think it can be any diseases… diabetes makes us susceptible to all the other diseases” (Men - adult group)

#### 3.1.4 A Disease with ‘No’ Cure

Alternative medicine was a commonly trusted and prescribed treatment for diabetes in the communities. The participants mentioned that even diabetic patients from high socioeconomic positions were struggling to search for alternative diabetes medication. The participants discussed the issue of difficulties in finding medicine to cure diabetes. Those who had diabetes themselves mentioned their willingness to try any kind of alternatives that were believed to cure diabetes. However, the participants also mentioned how they were afraid that alternative medication would cause complications and make the condition worse. Diabetes was viewed as a disease with “no way out except death”.

“It seems like entering the wilderness because the medical world has not found a cure for diabetes” (Men – old group)

“If it is hypertension, we can just boil some herbs to lower the tension… but for sugar disease it is difficult… it may cause complications…” (Women – adult group)

#### 3.1.5 A Disease for ‘The Wealthy’

Diabetes was mentioned as a disease for ‘the wealthy’ in the community for several reasons. Wealthy people were assumed to be physically inactive because of their more sedentary nature of work. The wealthy could afford hiring a maid for housework. This was also related to the perception that “diabetics are usually lazy as they do almost no physical work”.

“We haven’t heard of any poor people having sugar disease, it’s usually those in the middle and upper-class. Maybe… wealthy people eat tasty food but don’t want to work… so their laziness (lack of activity) will cause sugar disease” (Women – young group)

The participants also mentioned how being wealthy implied having a wide variety of food options. They argued that wealthy people were ‘greedy’. Wealthy people were viewed as always eating tasty food as much as they liked as often as they wanted and that this brought on diabetes. Participants did not hesitate to raise these arguments even when they knew that one of the group members had diabetes.

“Her weight was 65kg! (Referring to one diabetic participant) She used to eat a lot… hahaha (laughing). We as her neighbours know that in her family the eating habit was amazing! They eat whatever they want; they (can afford to) buy fish, chicken, meat…anything they like… she likes to eat ‘Gule’ (a slow cooked meat with curry and coconut milk gravy) and only a little vegetable… so all the neighbours assume that she is sick because of her ‘big’ eating habit…” (Women – adult group)

*“Most diabetics are usually lazy… their activity does not produce sweat… not like us farmers… we sweat a lot”*.

“Yeaaa … those who work with less energy … just like him … (referring to one of the diabetic participants) … he does not work like us … that’s what we see” (Men – adult group)

In addition to their perception about diabetes as a disease for the wealthy, participants also perceived diabetes as a “high cost disease”, which makes poor people feel helpless about it. Its chronic nature requires daily management and treatments that were considered troublesome and expensive. In addition, diabetes care burdens all of the family members, especially when the children no longer stay with the parents. Some participants with diabetes in the family mentioned this problem.

“My mother was a diabetics, when we still had money we gave her medication, but after a while we ran out of money, then… She died… They (refer to medical practitioner) still do not have a cure for diabetes…” (Women – old group)

This condition was perceived as worse for diabetes patients from poor communities because they did not have sufficient resources to obtain the required medicines. The participants argued that only the wealthy have the chance of having a long life with diabetes, and that the poor must be ready to die.

“He (a diabetic) has money…that means he can afford to buy injections so he has a long life, but for people who don’t have money, the disease becomes severe…If we do not have money, then we will die very soon” (Women – old group)

### 3.2 Content Area 2, Perception about Risk Factors: “Unrealistic Optimism in Diabetes Risk Factors Perception”

The overall theme identified within this content area was that the perceptions on risk factors are characterized by “unrealistic optimism”. This theme was developed based on four categories as described below.

#### 3.2.1 Heredity and Sugar is the Main Risk

All of the participants recognized heredity as one of the risk factors for diabetes. The participants directly referred ‘sugar disease’ as a ‘heredity’ disease. Some participants argued that a heredity disease can also be “spread” through contact with the diabetic wounds (like an infectious disease) among family members with the “same blood or gene”. The participants believed that heritable factors were necessary to get diabetes. Thus, most participants believed that they could not develop diabetes because they did not have family members with diabetes.

“If the grandparents had diabetes, the kids or grandchildren will also have diabetes” (Men – old group)

“It’s impossible for me to have diabetes because I have no family with diabetes” (Men – young group)

Most of the participants believed that sugar disease could be caused by high consumption of sugar in drinks (tea, coffee, and syrups) and foods.

“I should reduce my sugar intake by not eating too much rice because it is sweet (to prevent diabetes). We should avoid all foods that contain sugar.” (Women – old group)

“Drinking coffee can be related to sugar disease because it contains sugar, cigarettes do not contain sugar.” (Men – young group)

#### 3.2.2 ‘Modern’ Lifestyle Is a Risk

Most of the participants were aware that unhealthy food and lifestyles were risk factors for diabetes. They agreed that most diabetics were fat, lazy, and used to eat only tasty food (referring to large quantities of meat or instant ‘modern’ food). Based on that, they argued that because they were just villagers that they were spared from the disease. They argued that they could not afford to eat meat or ‘modern’ food (i.e. burgers, fried chicken) every day, and that they got enough physical activity through farming activities. Participants argued that only ‘the wealthy’ could afford the ‘modern’ lifestyle so they are the ones at risk.

“I think…it is not possible for people like me to have diabetes. It is rare for a skinny person to have sugar disease. Most people with sugar disease are the fat ones” (Men - young group)

“A less physically active person is usually a lazy person…and this person is often stressed, so he is the one at risk…” (Women – old group)

Older participants stated that they used to live a healthier life compared to the younger generation that lives a modern life. The elderly claimed that they ate vegetables that they grew without any pesticides and cooked with pure herbs and spices in their traditional life. They never knew or consumed fast food except boiled vegetables that were freshly picked. The elderly argued that the modern lifestyle of the young generation makes them prone to illness.

*“This (modern) young generation seems to be more careful in terms of food, but are somehow more likely to consume instant food and use instant seasoning on food”*.

“The older generation do not know pesticides, preserved food, or preservatives…nowadays there are a lot of preservatives in the food…” (Women – old group)

#### 3.2.3 Older Age Increases Risk

The younger and older age groups had different perceptions on how age could be a risk factor for diabetes. The younger group felt that they were ‘immune’ to diabetes because of their youth or just because they were skinny.

“Older people, especially those who are fat, get sugar disease more easily… It is not possible for me to have diabetes…because I am young… hehehe (laughing)” (Men – young group)

The older participants accepted the view that being older meant that they have to be more cautious about their health, but they disagreed with the idea that diabetes only affected older people. Their wider social relations and connections allowed them to observe that diabetes affected both older and younger people in their villages.

“Older age has nothing to do with sugar disease…” (Men – old group)

“My friend had diabetes when she was 26 years old…” (Women – old group)

#### 3.2.4 Ambiguity in Smoking Risk

Although smoking is a big issue in Indonesia, the men and women in the FGDs had different perceptions on how smoking is related to diabetes. All men, including those who did not smoke, disagreed that smoking is a risk factor for diabetes. They argued that smoking was only related to lung disease, cancer, heart disease, and pregnancy disorders as mentioned in the health warnings on the cigarette pack. The participants believed that diabetes, which was seen only as sugar disease, was only caused by heredity factors or high sugar consumption. Some of the groups agreed, “It is better not to eat than to not smoke”.

Though they were aware of the warning signs on the cigarette pack, the male participants were not worried about the health effects of cigarettes and admitted that they continued smoking. The men’s groups also stated that if smoking really causes diabetes they would protest against the government for not mentioning it on the pack.

“In advertisements they said cigarettes cause cancer and heart attacks…but not diabetes” (Men – young group)

Most men participants also strongly argued for and defended the notion that smoking has positive health benefits. Because most of the participants were smokers, they compared their health to other participants who did not smoke but often developed a cough or other disease. The smokers believed that they had better health compared to non-smokers.

“I think nicotine will help our body to fight diabetes. Our elders are heavy smokers and they use a traditional way of smoking (using the dried tobacco leaves), which may have a lot of nicotine, but they were seldom sick, and they are very healthy! Smoking even prevents stroke because our lips become warm when we smoke and it will prevent stroke (which causes paralysis in the lips)” (Men – adult group)

On the other hand, women participants showed strong disagreement with smoking behaviour, even though some of them were not sure whether smoking is a risk factor for diabetes. There was a strong social stigma attached to women smoking in the community, and most of them stated that they had never and would never try smoking. Smoking was viewed as a ‘manly’ activity that was only allowed for men. In some Indonesian communities, women would be viewed as ‘not a good woman’ if they would have smoked.

“Smoking is very dangerous…it causes health problems, lungs, heart disease, and disturbs pregnancies…I am not sure about diabetes…I haven’t heard about that …” (Women – adult group)

“I will never try to smoke…” (Women – young group)

At the same time, the women also felt helpless about the smoking behaviour of their spouse or family member (i.e. father or brother). Most of the women participants did not work and were dependent on their spouse for a living. They argued that because the men were the ones who earned money, they had the right to decide what they will spend the money on and the wife cannot interfere.

“Men earn their money so they can spend their money on smoking”

“Our husbands are heavy smokers and it is difficult…(to stop them)” (Women – young group)

## 4. Discussion

### 4.1 Negative Perceptions towards the Disease Lead to Negative Attitudes towards the Diabetics

Our results showed that the participants perceives diabetes as ‘sugar’ disease or ‘sweet-pee’ disease, based on their perceptions of the ‘causes’ and ‘symptoms’ of diabetes, respectively. The term ‘diabetes’ is seen as a lavish name with more terrible complications compared to the term ‘sugar’ disease. It might be natural for the community to view a disease negatively because no individual wants to suffer from all of the negative consequences of being sick. Our findings regarding the poor perceptions towards diabetes were similar to those that have been discussed elsewhere in diverse settings such as Iran, the US, and Kenya using both quantitative and qualitative methods (Abdoli et al., 2012; Coronado et al., 2004; Maina et al., 2010; Tessaro et al., 2005). Similarly to our participants, the participants in these previous studies believed that diabetes was a heritable disease, and that an unhealthy lifestyle including poor diet and lack of exercise were the main risk factors. Almost all study participants in all of the settings viewed diabetes as a ‘terrifying’ disease. However, in the above studies, the participants did not use another term for diabetes (e.g. ‘sugar disease’) like we found in our study.

Helman (2007) points out that how a disease is perceived in the community depends on its recognition as ‘being something abnormal’. In our FGDs, diabetes was recognized when it caused unhealed wounds that led to amputations, thus signifying an abnormal state. Diabetes was perceived as a horrible disease because it causes severe wounds that are difficult to heal and, in the end, death.

As a result of poor perceptions of the disease, an associated negative attitude towards diabetics is clearly evident in the community. In one of the FGD discussions, one participant directly stated “this person is lazy, that is why he has diabetes” to another participant who had diabetes. We observed that this harsh statement was disturbing for the diabetic, but others in the group took it as a joke. One might argue that poor health literacy in the community might be a reason for such negative attitudes. People with poor health literacy frequently hold poor health beliefs and attitudes and this leads to poor health decisions (Berkman et al., 2011; von Wagner et al., 2009). The participants in our study seemed to believe that they knew ‘almost everything about diabetes’ even though some of their knowledge was incorrect and misleading, and they shared a common view and attitude of ‘blaming the victim’.

### 4.2 Unrealistic Optimism in Perceptions of Risk Factors

In our study, participants were able to recognize some of the relevant risk factors for diabetes and were also able to recognize and give rational arguments against the false examples (i.e. x-rays and pets). Diabetes was viewed as a ‘familial disease’ that is inherited through the generations and only attacks ‘the wealthy’ who are usually ‘lazy’ (have low physical activity) and ‘greedy’ (have poor eating behaviour). Other risk factors, such as smoking, fast food, low fruit and vegetable consumption, stress and anti-hypertensive medication were argued as having only indirect effects on developing diabetes. However, most of these risk factors that were viewed as being associated with ‘modern’ or ‘western’ lifestyles have become more prevalent in the rural Indonesian community (Ng, 2006) most probably as a consequence of globalization. The traditional and most healthy lifestyles are being abandoned while new ‘risky’ lifestyles are being adapted, and rural communities might have poor understanding on how these new risk factors can lead to NCDs. Consequently, a higher prevalence of diabetes is observed in low education and low socioeconomic communities (Jotkowitz et al., 2006).

Our results show an ambiguity with regards to perceptions about the risks of smoking for the development of diabetes. Almost all of the men in this study were smokers. They did not hesitate to smoke during our discussions even when discussing health problems, and they believed that smoking is related only to the diseases stated on the cigarette pack. In Indonesia, the cigarette pack contains the message “smoking can cause cancer, heart attack, impotence, and fetal and pregnancy disorders”. Indonesia faces problems in tobacco control policies. Both the smokers and the non-smokers in our study defended the ‘unhealthy’ smoking habit and argued that those who were not smoking were those who always got sick. As the fifth-largest producer and exporter of tobacco leaf globally, the Indonesian government claims that the tobacco industries contribute the largest source of income for the country. Indonesia has the third-highest rate of cigarette consumption in the world, and 67% of Indonesian men and 3% of Indonesian women are daily smokers. Smoking is more prevalent in rural areas (39%), the areas where people tend to be poor, compared to urban areas (33%) (WHO, 2012). For the prevention of diabetes (as well as other diseases) in rural Indonesia, interventions need to acknowledge the need for further awareness and knowledge about the multiple health risks caused by smoking.

In sum, our participants were found to hold an “unrealistic optimism” in the risk for developing diabetes. ‘Unrealistic optimism’, also known as ‘optimistic bias’, has been frequently observed in health psychology studies of risk perceptions. This concept attempts to explain how people fail to act because they assume a certain disease will occur in others, not themselves (Morrison & Bennett, 2009). Our FGD data shows that rural communities tend to be unrealistically optimistic when considering the risk for developing diabetes. They assume that they will be protected from diabetes due to their low body weight, lack of diabetic family members, and rural working lifestyle with a poor socioeconomic position. Chronic diseases such as diabetes were believed to affect mainly wealthy people and older people. Wealthy people were also seen as being able to cope with the expenses of managing the disease Furthermore, the participants stated that the disease is a result of unhealthy lifestyles and that the diabetics themselves are to be blamed. Our findings support the WHO report that myths in regards to chronic disease exist in the global community (WHO, 2005).

### 4.3 Understanding the Result Based on Existing Cultural Health Beliefs

Chronic NCD prevention and management is the focus of a large proportion of the therapeutic activities engaged in by healthcare practitioners. Yet people very often fail to follow the recommended actions, or the self-management protocols. This causes them to suffer from the disease and develop complications that they could otherwise avoid. A common question in the healthcare field is why this might be so.

Our findings can be discussed in light of two theoretical models, “the Health Belief Model” and “risk perception”. Morrison and Bennett defined health behaviour as “behaviour performed by an individual, regardless of their health status, as a means of protecting, promoting, or maintaining health” (Morrison & Bennett, 2009). This definition suggests that many people constantly try to be healthy and to protect themselves from any negative events such as accidents or diseases. Our study shows that diabetes is perceived as a scary disease that passes from generation to generation and is a result of an unhealthy lifestyle. The severity of the disease is well perceived, and the participants in our study were well aware of the terrible complications that can develop with this disease. Balancing healthy food intake with physical activity and avoiding fast food is believed to promote good health. At the same time, most participants were unrealistically optimistic as to their perceived susceptibility to the disease. They believed only those who have family with diabetes, the wealthy, the older, and the fatter will develop the disease. The men in the study were even more unrealistically optimistic in perceiving the effects of smoking as being good for their health.

In referring to the Health Belief Model, Jans and Becker (1984) state that preventive health behaviour is predicted by the following three sets of beliefs: perceived susceptibility, perceived severity, and perceived benefits or barriers (Bury & Gabe, 2004; Glanz et al., 2002). In our study, the participants believed that they would not develop the disease unless they have family members with diabetes (perceived susceptibility), they were aware of the disease and its complication (perceived severity), and they knew that exercise and healthy food would improve their health and were aware that if they developed the disease that it would be very costly (perceived benefits). The three sets of beliefs in the Health Belief Model are strongly influenced by how people define and experience health and illnesses (Albrecht et al., 2003). Culture may play a role in determining a community’s health by means of either improving health or causing disease. In Javanese culture, disease is only perceived if people *feel* or *see* the effect of the disease on the body. This is confirmed by an ethnographic study on Javanese culture that defines a disease symptom as *rasa* or *feeling* (Ferzacca, 2001). This might explain why most participants have unrealistic optimism about their own risk, because most of them stated that they did not *feel* or *see* the disease symptoms on them.

Lay theories about the cause of illness often postulate that illnesses can be caused by the individual, the natural world, the social world, the supernatural world, or a combination thereof (Helman, 2007). The experience of illnesses is affected not only by the individual’s perceptions but also by the community’s perceptions (Albrecht et al., 2003). Our study shows that the participants believe that the origin of diabetes lies within the individual. They believe that diabetes is caused by individual choices such as an unhealthy lifestyle, heredity, or their psychological or physical weakness, and, therefore, the responsibility falls on the ill individuals themselves. Furthermore, these perceptions can be psychologically harmful to the ill individuals when an unhealthy lifestyle or having an inherited disease is viewed as living a ‘sinful life’.

### 4.4 Implications for Diabetes Prevention

Recognizing specific health and illness experiences in different cultures is necessary to achieve successful health promotion or disease prevention programs. The design of prevention strategies should be sensitive to the local culture. Javanese people have a strong belief in maintaining “balance and harmony” in their family and social relations and in their personal health (Dewi et al., 2010). The concept of “balance and harmony” could be used when delivering the health message to this specific community. Tailoring health messages to the importance of balancing healthy food with physical activity would be one example. A variety of health promotion activities could be arranged together with local leaders to gain more participants. Combining the community gymnasium (physical activity) with healthy cooking demonstrations might also be one effective strategy. Health promotion should not be limited to interpersonal transmission of health information or media campaigns. Creating supportive environments and tailoring interventions together with people in the community will improve their health literacy and help them to have the confidence to act in their own best interests (Nutbeam, 2000).

### 4.5 Limitations of the Study

A limitation of this study is that it did not exclude respondents with diabetes experience (diabetic or having family with diabetes). The recruitment processes allowed everyone that met the aforementioned requirement to be included in the discussion group (*see section 2.1*). One might argue that the appearance of diabetes-experienced participants may have affected the other participant’s views. However, the authors did not feel that this condition affected the flow and content of the discussions since everyone could openly raise their thoughts and ideas, including the negative attitude towards the diabetics.

We had planned to conduct the FGDs following a scheduled date and time as informed in the invitation letter. Unfortunately, due to problems for some of the participants to attend at the given time, some of the FGDs could not be run on the scheduled time. This might somehow have affected the discussion flow, since the respondents who came late needed some time to catch up.

Given the nature and prerequisites for qualitative studies, the results of this study cannot be seen as representative of the whole Javanese population, but might reflect Purworejo District as well as other settings with a similar social context.

## 5. Conclusions

The findings of our study show that rural Indonesian communities have negative perceptions towards diabetes that result in negative attitudes towards the diabetics, and the diabetics themselves were blamed for their disease. Furthermore, people in these communities have an unrealistic optimism where it is only ‘others – not me’ that can develop the disease. These points to the importance of developing and delivering appropriate information on diabetes risk factors and prevention strategies and activities.

Effective prevention strategies need to be tailored to this specific population by understanding their health behaviour and conceptualizations of health and diseases. Integrating different professions, including chefs, physical trainers, and psychologists along with local leaders would be beneficial to improving the effectiveness of any prevention strategies.
